# Sevoflurane Promotes Neurodegeneration Through Inflammasome Formation in APP/PS1 Mice

**DOI:** 10.3389/fnins.2021.647136

**Published:** 2021-12-02

**Authors:** Guohua Li, Yu Wang, Fang Cao, Dawei Wang, Limin Zhou, Yanwu Jin

**Affiliations:** ^1^Department of Anesthesiology, The Second Affiliated Hospital of Shandong First Medical University, Tai’an, China; ^2^Department of Orthopedics, The Second Affiliated Hospital of Shandong First Medical University, Tai’an, China; ^3^Department of Obstetrics, The Affiliated Hospital of Qingdao University, Qingdao, China; ^4^Department of Anesthesiology, The Second Hospital, Cheeloo College of Medicine, Shandong University, Jinan, China

**Keywords:** sevoflurane (SEVO), Alzheimer’s disease (AD), NLRP3, BDNF, neurodegenaration

## Abstract

Sevoflurane (SEVO) is a highly fluorinated methyl isopropyl ether used as an inhalational anesthetic for general anesthesia. Previous studies have shown that SEVO may induce impaired memory and recognition ability and may be associated with neurodegenerative disease, e.g., Alzheimer’s disease (AD). However, the underlying mechanism remains unknown. Here, we used a mouse AD model, APP/PS1, to study the effects of SEVO on neurodegeneration occurring in AD. We found that SEVO exposure significantly impaired the spatial reference memory, sensorimotor, and cognitive function of the mice. Mechanistically, we found that SEVO induced formation of NOD-, LRR- and pyrin domain-containing protein 3 (NLRP3) inflammasome and its downstream caspase 1-mediated production of IL-1β and IL-18, which subsequently deactivated brain-derived neurotrophic factor (BDNF) to promote neurodegeneration. Together, these data suggest that NLRP3 inflammasome is essential for SEVO-induced AD.

## Introduction

Isoflurane and sevoflurane (SEVO) are the most widely used inhaled anesthetics, given their rapid induction and prompt recovery coupled with stimulation. However, these commonly used volatile anesthetics have been recently shown to exert neurotoxic effects, like brain injury (Zhang et al., 2015; Wang et al., 2018) and behavioral impairments (Zhao et al., 2018). SEVO is specially more and more recommended not to be used as a long-term anesthetic, likely due to its generation of catabolic compound A to mediate significant nephrotoxicity during anesthesia (Higuchi et al., 1998).

The detrimental effects of SEVO on neuronal cell integrity have been proposed in some previous studies. As early as 2009, exposure of newborn rats to SEVO has been shown to induce apoptotic neurodegeneration in the developing brain (Bercker et al., 2009). Moreover, SEVO exposure in 7-day-old rats was found to impair neurogenesis, induce neurodegeneration, and affect neurocognitive function (Fang et al., 2012). Later studies have found that some treatments may improve SEVO-induced neural injuries (Lei et al., 2013; Goyagi, 2019a,b). For the mechanistic studies, SEVO was shown to activate gamma-aminobutyric acid (GABA) to induce overexcitability of GABA receptors and subsequent inflow of extracellular calcium ions, which caused neurotoxicity and long-term cognitive impairment (Morris et al., 1982; Burgess et al., 2002; Fang et al., 2012). SEVO was also shown to suppress *N*-methyl-D-aspartate (NMDA) receptors to reduce extracellular glutamate levels and thus impaired synapse formation and intercellular connections of neuronal cells (Criswell et al., 2004). In addition, tau protein has been shown as a target for SEVO (Le Freche et al., 2012; Hu et al., 2013; Tao et al., 2014; Liu et al., 2017; Hou and Xiao, 2019; Yang et al., 2020; Yu et al., 2020; Zhang et al., 2020; Dong et al., 2021; Sun et al., 2021). Brain-derived neurotrophic Factor (BDNF) is a key neurotrophic factor that promotes synaptic plasticity as well as the survival and function of neuronal cells (Aarons et al., 2019; Bomba et al., 2019; Fransquet et al., 2020). Challenge with SEVO decreased BDNF levels in neuronal cells, which led to compromised spatial memory ability of rats in a serine/threonine acid kinase (Akt)/glycogen synthase kinase 3β (GSK-3β) signaling-dependent manner (Chen et al., 2015). However, to date, the exact molecular mechanisms that underlie the neurotoxic effects of SEVO are still elusive, which prevents effective interference to prevent neurotoxicity.

Activation of the immune system and inflammation play an essential role in cognitive impairment (Walsh et al., 2014). NOD-, LRR-, and pyrin domain-containing protein 3 (NLRP3) is the best characterized inflammasome and has been implicated in the development of neurodegenerative diseases (Albornoz et al., 2018). Very recently, it was shown that Chikusetsu saponin IVa may alleviate SEVO-induced neuroinflammation and cognitive impairment by blocking NLRP3, likely through reducing apoptosis of neuronal cells (Shao et al., 2020). However, the exact mechanisms were still unknown, especially the involvement of BDNF.

Here, we used a mouse AD model, APP/PS1, to study the effects of SEVO on neurodegeneration occurring in AD. We found that SEVO exposure significantly impaired the spatial reference memory, sensorimotor, and cognitive function of the mice. Mechanistically, we found that SEVO induced formation of NLRP3 inflammasome and its downstream caspase 1-mediated production of IL-1β and IL-18, which subsequently deactivated BDNF to promote neurodegeneration.

## Materials and Methods

### Protocol Approval

All the experimental methods including animal experiments have been approved by the research committee of the second hospital of Shandong University.

### Animals

All experiments were performed in strict accordance with the Care and Use of Laboratory Animal Guideline, issued by the second hospital of Shandong University. APPswe/PSEN1dE9 (APP/PS1) (Jankowsky et al., 2004) transgenic mice were purchased from Jackson Labs (Bar Harbor, ME, United States). The APP/PS1 mice were individually housed under a 12-h light–dark cycle (temperature: 24 ± 2°C; humidity: 46 ± 4%) with *ad libitum* access to water and food. These mice typically developed significant AD-associated pathological features and behavioral disorders at 6 months of age. In order to assess the effects of SEVO, we exposed the mice at 3 months of age in 5% SEVO or air with an identical flow rate of 1.2 L/min for 8 h. The mice were then kept for another 2 months before analysis. MCC950 (Cayman Chemical, Ann Arbor, MI, United States) treatment was performed at a dose of 4.5 mg/kg in 50 μl *via* tail vein immediately before exposure to SEVO and every 2 h during the exposure, altogether five times, as described (Coll et al., 2015). The control mice received dimethyl sulfoxide (DMSO), the dissolving medium for MCC950.

### Flow Cytometry

For purification of neuronal cells, the brain tissue was dissociated into single cells by digestion with 0.25% trypsin (Sigma-Aldrich) for 35 min then fixation in 2% formalin before incubation with flourescein isothiocyanate (FITC)-conjugated anti-NeuN antibody (Novus Biologicals, CO, United States). The flow charts were generated using Flowjo software (Flowjo LLC, Ashland, OR, United States).

### Quantitative PCR

Total RNA was extracted using RNeasy mini kit (Qiagen, Hilden, Germany) and then reversely transcribed to complementary DNA (cDNA) using miScript II RT Kit (Qiagen). Quantitative PCR was performed in duplicates with QuantiTect SYBR Green PCR Kit (Qiagen). All primers were purchased from Qiagen (the sequences of these primers are not open to customers perhaps due to trade secret). Values of genes were determined by sequential normalization to glyceraldehyde 3-phosphate dehydrogenase (GAPDH) and the experimental controls.

### Western Blot and ELISA

The isolated cells or tissue were homogenized in radioimmunoprecipitation assay (RIPA) protein lysis buffer (Thermo scientific) to extract proteins, the concentration of which was determined using a bicinchoninic acid (BCA) protein assay kit (Bio-rad, Beijing, China). Western blot was done as routine, using primary antibodies including rabbit anti-BDNF, anti-NLRP3, anti-caspase 1, and anti-GAPDH (1:1,000 for all, Abcam, Cambridge, MA, United States). The secondary antibody was horseradish peroxidase (HRP)-conjugated anti-rabbit (Jackson ImmunoResearch Labs, West Grove, PA, United States). The presentative blot images were randomly selected from five individuals. NIH ImageJ software (Bethesda, MA, United States) was used for image acquisition and densitometric analysis of the gels. ELISA was done using mouse Aβ, p-Tau, IL-1β, and IL-18 ELISA kits (R&D System, Los Angeles, CA, United States).

### Immunohistochemistry and Quantification

Hemibrains (8-h fixation with 10% formalin) were processed and embedded in paraffin. Serial sections were cut at 5 μm, mounted and rehydrated according to standard protocols. For immunostaining, standard procedures with peroxidase-labeled streptavidin and 3,3′-diaminobenzidine (DAB) chromagen were applied. The immunostaining was done using rabbit anti-Aβ antibody (1:100, MD Millipore, Billerica, MA, United States), goat anti-phosphorylated Tau (p-Tau) (1:200, Santa Cruz Biotechnology, Dallas, Texas, United States), or rabbit anti-PHF1 antibody (1:250, Abcam) antibodies. To quantitate neuronal cell loss, PHF-1+ neuronal positive cells were counted for density (cells/mm^3^) in a systematic random fashion throughout the CA1 region of the hippocampus.

### Behavioral Test

Spatial reference memory was measured using the Morris water-maze test. Mice were handled 60 s/day for 10 days during the 2 weeks prior to the real test. Pre-handling was designed to condition the mice to manipulations which would be experienced during introduction and removal from the testing pool and included a 20-s exposure to water at a depth of 1 cm. Mice were tested at the age of 5 months. During the test, mice received visible platform training for 3 days, six trials per day, and hidden platform training for 6 days, three trials per day. Four probe trials of 30-s duration were performed 20 h after the hidden training trials. The mean target quadrant occupancy of all four probes was calculated.

For sensorimotor and cognitive test, the composite score comprised beam walk and Bederson score (circling bias, forelimb retraction, hindlimb reflex, and resistance to push). For forelimb and hindlimb retraction, the ability of the animal to replace the limb after being displaced laterally was measured (2 for immediate replacement, 1 for replacement after a minute, or 0 for no replacement). Resistance to push received a 1 if it did resist or 0 if there was no resistance to push. Beam walk was graded on a 7-point scale. Composite neurological score comprised both the Bederson score and beam walking score on a 1–14 scale. For novel object recognition, animals were habituated for 10 min in the testing apparatus for 4 days prior to baseline testing. On testing days, the mice participated in three phases: acclimation, familiarization, and novel. In the acclimation phase, a mouse was allowed to explore two identical objects placed equidistant from the walls with 20 cm between the objects for 5 min during the familiarization phase. After a 15-min delay in their home cage, the animal was allowed to explore a novel object paired with the familiar object for 5 min. Animals were started in the center of the testing apparatus for each session. The time spent exploring each object was recorded, and recognition index [RI; total time (s) spent exploring novel object (TN) divided by the total time spent exploring both novel and familiar (TF) objects: RI = TN/(TN + TF)] and discrimination index [DI; DI = (TN - TF)/(TN + TF)] were calculated for the familiarization and novel phases. For novel social recognition, mice were habituated to the three-chamber box for 3 days prior to testing for 10 min. Conspecifics were habituated to baskets for 15 min the day before testing. On the day of testing, the test mouse was acclimated to the testing apparatus for 5 min with empty baskets on the outer chambers. In the first phase, animals were allowed to explore the three-chamber box with one empty basket and one basket with a conspecific (C1) for 5 min. After a 15-min delay in their home cage, animals were placed back in the testing apparatus with C1 under one basket and a second conspecific (C2) for 5 min. For half the experimental rats C1 remained on the same side of the box in both trials, while the other half was placed in the other chamber for the second trial. Time spent in each chamber and time spent exploring the conspecific/empty basket was reported.

### Statistics

GraphPad Prism 6 (GraphPad Software, San Diego, CA, United States) was used for statistical analysis. Analysis was done by one-way ANOVA with a Bonferroni correction, followed by Fisher’s exact test upon necessity. All values are depicted as mean ± standard error from 5 to 10 individuals (N represents animal number per experimental group or number of repeats in the culture studies) and are considered significant if *p* < 0.05.

## Results

### SEVO Impairs Spatial Reference Memory, Sensorimotor, and Cognitive Function of AD-Prone Mice

**FIGURE 1:**
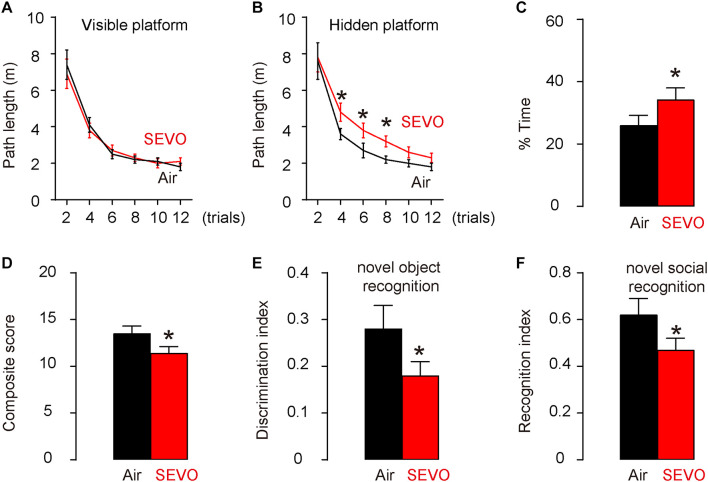
SEVO impairs spatial reference memory, sensorimotor, and cognitive function of AD-prone mice. AD-prone APP/PS1 mice, 3 months of age, were randomly selected into two groups of 10 each. The SEVO group was exposed to 5% SEVO for 8 h, while the control group was exposed to normal air (Air). Afterward, the mice were kept for another 2 months before assessment of their behavioral changes. **(A–C)** Morris water maze test for assessing spatial reference memory. **(A)** Path length in the visible platform phase. **(B)** Path length in the hidden platform phase. **(C)** Target quadrant occupancy. **(D–F)** Sensorimotor and cognitive test. **(D)** Composite score. **(E)** Novel object recognition task. **(F)** Novel social recognition task. *p < 0.05. *N* = 10. N represents mouse number per experimental group.

AD-prone APP/PS1 mice, 3 months of age, were randomly selected into two groups of 10 each. The SEVO group was exposed to 5% SEVO for 8 h, while the control group was exposed to normal air (Air). Afterward, the mice were kept for another 2 months before assessment of their behavioral changes. These mice do not develop very significant alterations in behavioral tests and in pathology at this age (5 months of age) (Ferguson et al., 2013). Morris water maze test for assessing spatial reference memory showed that SEVO-exposed mice exhibited no difference in the path length in the visible platform phase (Figure 1A) but required significantly longer path length in the hidden platform phase (Figure 1B) and significantly increased target quadrant occupancy (Figure 1C), compared to air-control mice. For sensorimotor and cognitive test, SEVO-exposed mice exhibited more severe sensorimotor deficits in composite score measurements, compared to air-control mice (Figure 1D). Cognition was assessed by novel object recognition task (Figure 1E) and novel social recognition task (Figure 1F), both showing more impaired hippocampal-dependent cognition in SEVO-exposed mice, compared to air-control mice. Together, these data suggest that SEVO impairs spatial reference memory, sensorimotor, and cognitive function of AD-prone mice.

### SEVO Increases Loss of Neuronal Cells, Aβ Deposition, and p-Tau Formation in the Brain of APP/PS1 Mice

**FIGURE 2:**
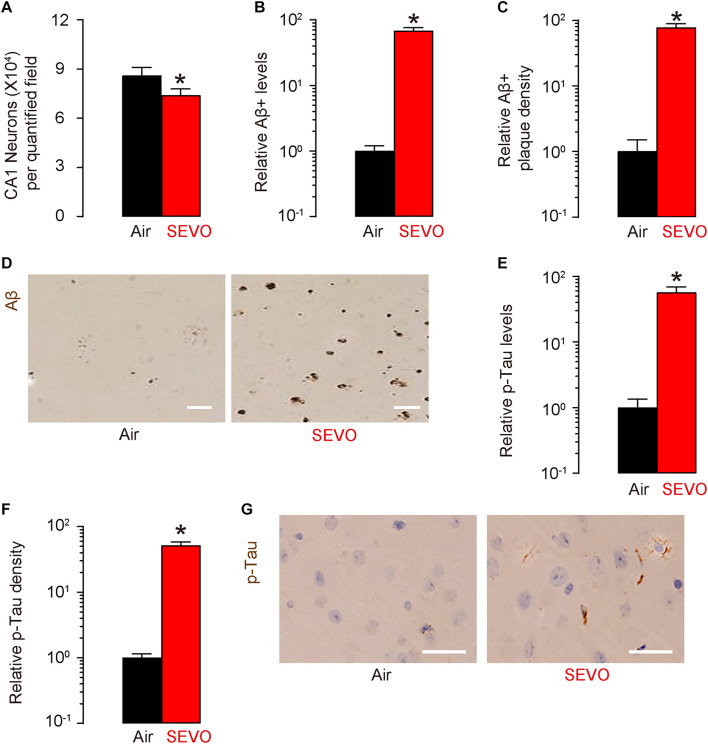
SEVO increases loss of neuronal cells, Aβ deposition, and p-Tau formation in the brain of APP/PS1 mice. **(A)** Neuron loss in the hippocampus region of APP/PS1 mice at sacrifice (2 months after SEVO/Air exposure and at 5 months of age). **(B)** ELISA for Aβ levels in hippocampus from SEVO-exposed mice, compared to air-control mice. **(C,D)** Aβ plaque density in the hippocampus region in SEVO-exposed mice, compared to air-control mice, shown by quantification **(C)** and by representative images **(D)**. **(E)** ELISA for p-Tau levels in hippocampus from SEVO-exposed mice, compared to air-control mice. **(F,G)** p-Tau density in the hippocampus region in SEVO-exposed mice, compared to air-control mice, shown by quantification **(F)** and by representative images **(G)**. *p < 0.05. *N* = 10. N represents mouse number per experimental group. Scale bars are 20 μm.

At sacrifice (2 months after SEVO/Air exposure and at 5 months of age), we quantified loss of neuronal cells in the hippocampus region of the APP/PS1 mice. We detected significantly less neuronal cells in SEVO-exposed mice, compared to air-control mice (Figure 2A). Next, we analyzed the Aβ levels by ELISA in the hippocampus region, showing significantly higher Aβ in SEVO-exposed mice, compared to air-control mice (Figure 2B). Moreover, we detected significantly higher Aβ plaque density in the hippocampus region in SEVO-exposed mice, compared to air-control mice, shown by quantification (Figure 2C) and by representative images (Figure 2D). We also analyzed the p-Tau levels by ELISA in the hippocampus region, showing significantly higher p-Tau in SEVO-exposed mice, compared to air-control mice (Figure 2E). Moreover, we detected significantly higher p-Tau density in the hippocampus region in SEVO-exposed mice, compared to air-control mice, shown by quantification (Figure 2F) and by representative images (Figure 2G). Together, these data suggest that SEVO increases loss of neuronal cells, Aβ deposition, and p-Tau formation in the brain of APP/PS1 mice and thus promotes AD progression.

### SEVO Reduces BDNF and Increases NLRP3 Inflammasome in Neuronal Cells

**FIGURE 3:**
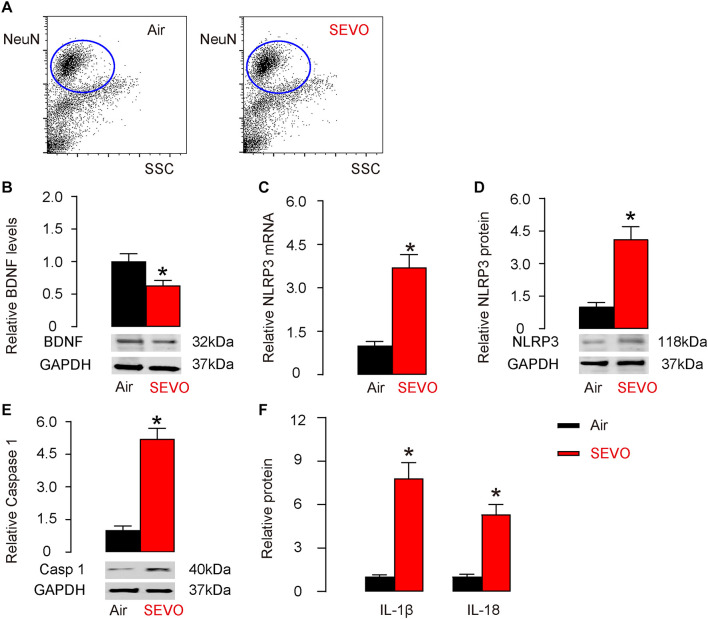
SEVO reduces BDNF and increases NLRP3 inflammasome in neuronal cells. **(A)** Representative flow charts for isolation of NeuN-positive neuronal cells from mouse hippocampus by flow cytometry. **(B)** Western blot for BDNF in neuronal cells from SEVO-exposed mice, compared to those from air-control mice. **(C,D)** RT-qPCR **(C)** and Western blot **(D)** for NLRP3 in neuronal cells from SEVO-exposed mice, compared to those from air-control mice. **(E)** Western blot for caspase 1 in neuronal cells from SEVO-exposed mice, compared to those from air-control mice. **(F)** ELISA for IL-1β and IL-18 in neuronal cells from SEVO-exposed mice, compared to those from air-control mice. *p < 0.05. *N* = 10. N represents mouse number per experimental group.

We studied the mechanisms that underlie the accelerating effects of SEVO on neurodegeneration occurring in AD. BDNF is a key neurotrophic factor that promotes the survival of existing neurons and is essential for antagonizing neurodegeneration. NeuN-positive neuronal cells were purified from mouse hippocampus by flow cytometry (Figure 3A). We examined BDNF levels by Western blot and detected significantly lower BDNF levels in neuronal cells from SEVO-exposed mice, compared to those from air-control mice (Figure 3B). A recent study has demonstrated the negative regulation of BDNF by NLRP3 inflammasome (Ward et al., 2019). Thus, we examined the levels of NLRP3. We detected significant increases in NLRP3 mRNA (Figure 3C) and protein (Figure 3D) in neuronal cells from SEVO-exposed mice, compared to those from air-control mice. Moreover, the downstream factors of NLRP3, caspase 1 (Figure 3E), IL-1β, and IL-18 (Figure 3F) all increased in neuronal cells from SEVO-exposed mice, compared to those from air-control mice. Thus, SEVO reduces BDNF and increases NLRP3 inflammasome in neuronal cells.

### Suppression of NLRP3 Attenuates Adverse Effects of SEVO on Spatial Reference Memory, Sensorimotor, and Cognitive Function in Mice

**FIGURE 4:**
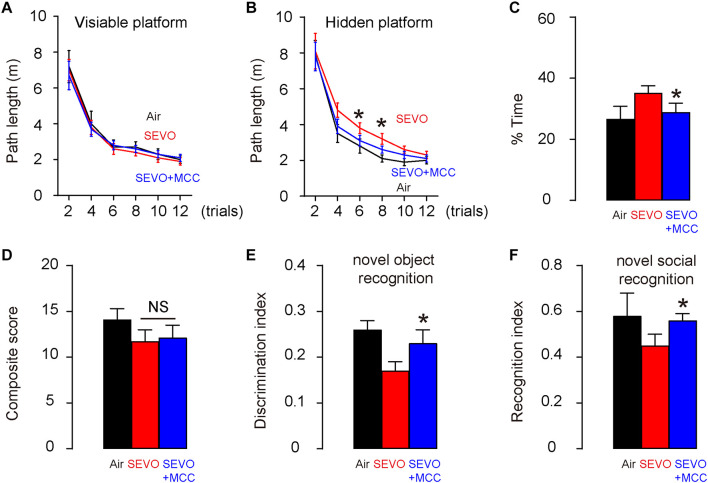
Suppression of NLRP3 attenuates adverse effects of SEVO on spatial reference memory, sensorimotor, and cognitive function in AD-prone mice. Five interferences with the NLRP3 inhibitor, MCC950, at an interval of 2 h in between, was done, starting immediately before the SEVO/Air exposure (SEVO+MCC). The control mice received DMSO, the dissolving medium for MCC950 (SEVO). All AD-prone APP/PS1 mice of 3 months of age were then exposed to 5% SEVO for 8 h. Afterward, the mice were kept for another 2 months before assessment of their behavioral changes. **(A–C)** Morris water maze test for assessing spatial reference memory. **(A)** Path length in the visible platform phase. **(B)** Path length in the hidden platform phase. **(C)** Target quadrant occupancy. **(D–F)** Sensorimotor and cognitive test. **(D)** Composite score. **(E)** Novel object recognition task. **(F)** Novel social recognition task. *p < 0.05 (SEVO+MCC vs. SEVO). *N* = 10. N represents mouse number per experimental group.

To figure out whether SEVO may promote AD-associated neurodegeneration through activation of NLRP3 inflammasome, we did five interferences with NLRP3 inhibitor, MCC950, with an interval of 2 h in between, starting immediately before the SEVO/Air exposure (SEVO+MCC). The control mice received DMSO, the dissolving medium for MCC950 (SEVO). All AD-prone APP/PS1 mice of 3 months age were then exposed to 5% SEVO for 8 h. Afterward, the mice were kept for another 2 months before assessment of their behavioral changes. Morris water maze test for assessing spatial reference memory showed that SEVO+MCC mice exhibited no difference in the path length in the visible platform phase (Figure 4A) but required a significantly shorter path length in the hidden platform phase (Figure 4B) and significantly decreased target quadrant occupancy (Figure 4C), compared to SEVO mice. For sensorimotor and cognitive test, SEVO+MCC mice exhibited no difference in the sensorimotor deficits in composite score measurements, compared to SEVO mice (Figure 4D). However, cognition was assessed by novel object recognition task (Figure 4E) and novel social recognition task (Figure 4F), both showing significantly attenuated impaired hippocampal-dependent cognition in SEVO+MCC mice, compared to SEVO mice. Together, these data suggest that suppression of NLRP3 attenuates adverse effects of SEVO on spatial reference memory, sensorimotor, and cognitive function in AD-prone mice.

### Suppression of NLRP3 Attenuates SEVO-Mediated Loss of Neuronal Cells, Aβ Deposition, and p-Tau Formation in the Brain of APP/PS1 Mice

**FIGURE 5:**
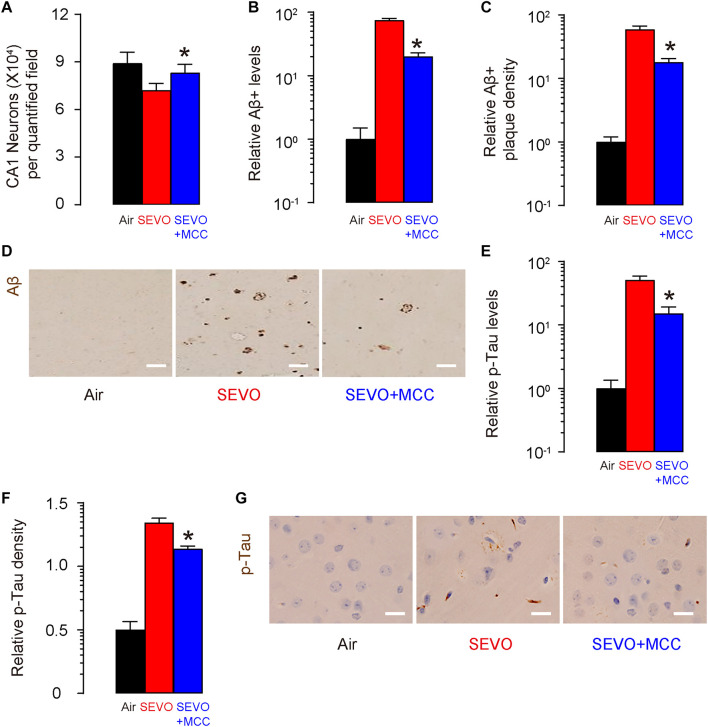
Suppression of NLRP3 attenuates SEVO-mediated loss of neuronal cells, Aβ deposition, and p-Tau formation in the brain of APP/PS1 mice. **(A)** Neuron loss in the hippocampus region of APP/PS1 mice at sacrifice (2 months after SEVO exposure and MCC/DMSO treatment and at 5 months of age). **(B)** ELISA for Aβ levels in hippocampus from SEVO+MCC mice, compared to SEVO mice. **(C,D)** Aβ plaque density in the hippocampus region in SEVO+MCC mice, compared to SEVO mice, shown by quantification **(C)** and by representative images **(D)**. **(E)** ELISA for p-Tau levels in hippocampus from SEVO+MCC mice, compared SEVO mice. **(F,G)** p-Tau density in the hippocampus region in SEVO+MCC mice, compared to SEVO, shown by quantification **(F)** and by representative images **(G)**. *p < 0.05 (SEVO+MCC vs. SEVO). *N* = 10. N represents mouse number per experimental group. Scale bars are 20 μm.

At sacrifice (2 months after SEVO exposure and MCC/DMSO treatment and at 5 months of age), we quantified loss of neuronal cells in the hippocampus region of these mice. We detected significantly more neuronal cells in SEVO+MCC mice, compared to SEVO mice (Figure 5A). Next, we analyzed the Aβ levels by ELISA in the hippocampus region, showing significantly lower Aβ in SEVO+MCC mice, compared to SEVO mice (Figure 5B). Moreover, we detected significantly lower Aβ plaque density in the hippocampus region in SEVO+MCC mice, compared to SEVO mice, shown by quantification (Figure 5C) and by representative images (Figure 5D). We also analyzed the p-Tau levels by ELISA in the hippocampus region, showing significantly lower p-Tau in SEVO+MCC mice, compared to SEVO mice (Figure 5E). Moreover, we detected significantly lower p-Tau density in the hippocampus region in SEVO+MCC mice, compared to SEVO mice, shown by quantification (Figure 5F) and by representative images (Figure 5G). Together, these data suggest that suppression of NLRP3 attenuates SEVO-mediated loss of neuronal cells, Aβ deposition, and p-Tau formation in the brain of APP/PS1 mice.

### Suppression of NLRP3 Increases BDNF in Neuronal Cells

**FIGURE 6:**
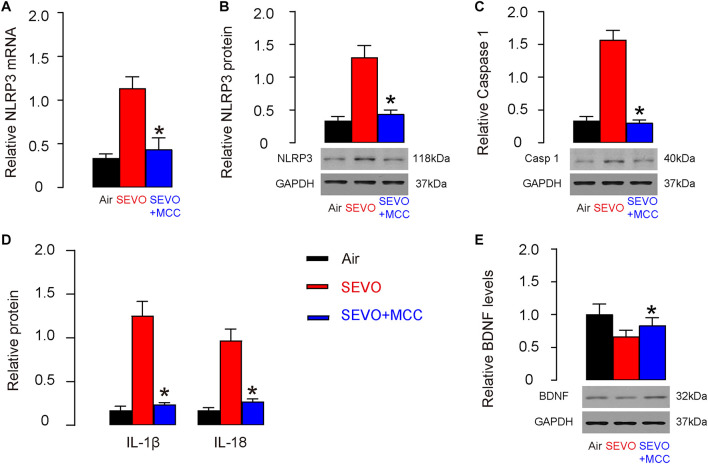
Suppression of NLRP3 increases BDNF in neuronal cells. **(A,B)** RT-qPCR **(A)** and Western blot **(B)** for NLRP3 in neuronal cells from SEVO+MCC mice, compared to those from SEVO mice. **(C)** Western blot for caspase 1 in neuronal cells from SEVO+MCC mice, compared to those from SEVO mice. **(D)** ELISA for IL-1β and IL-18 in neuronal cells from SEVO+MCC mice, compared to those from SEVO mice. **(E)** Western blot for BDNF in neuronal cells from SEVO+MCC mice, compared to those from MCC mice. *p < 0.05 (SEVO+MCC vs. SEVO). *N* = 10. N represents mouse number per experimental group.

Next, NeuN-positive neuronal cells were purified from mouse hippocampus by flow cytometry. We detected significant reduction in NLRP3 mRNA (Figure 6A) and protein (Figure 6B) in neuronal cells from SEVO+MCC mice, compared to those from SEVO mice. Moreover, the downstream factors of NLRP3, caspase 1 (Figure 6C), IL-1β, and IL-18 (Figure 6D) all decreased in neuronal cells from SEVO+MCC mice, compared to those from SEVO+MCC mice. These data confirmed the inhibitory effects of MCC950 on NLRP3. Next, we examined BDNF levels by Western blot and detected significant increases in BDNF levels in neuronal cells from SEVO+MCC mice, compared to those from SEVO mice (Figure 6E). Thus, suppression of NLRP3 increases BDNF in neuronal cells.

### Bioinformatic Analysis Shows That SEVO Upregulates NLRP3 in Brain

**FIGURE 7:**
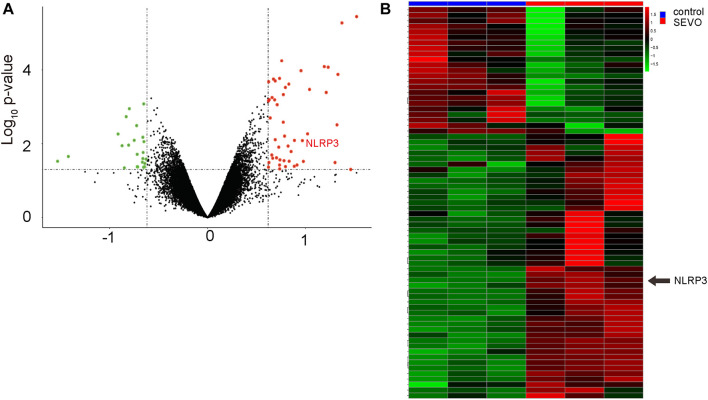
Bioinformatic analysis shows that SEVO upregulates NLRP3 in brain. **(A,B)** Data from GSE141242 showed that SEVO indeed increased NLRP3 levels in brain, by a volcano map **(A)** and by a heat map **(B)**.

Finally, we searched a published database for the evidence to support the effects of SEVO on NLRP3. Data were obtained from GSE141242, showing that SEVO indeed increased NLRP3 levels in brain, by a volcano map (Figure 7A) and by a heat map (Figure 7B).

## Discussion

Wild-type mice are not used in the current study since they are not susceptible to SEVO compared to APP/PS1 mice, a widely used mouse model specifically generated for studying neurodegeneration in AD (Jankowsky et al., 2004). Therefore, the neurotoxic effects of SEVO are difficult to be detected in wild-type mice but could be detected in APP/PS1 mice due to their susceptibility for neurodegeneration. Hence, the results obtained on the neurotoxic effects of SEVO in this study may not reflect a general situation but suggest that SEVO exacerbates a neurodegenerative process already underway.

The AD-prone APP/PS1 mice typically develop detectable features at 6 months of age (Jankowsky et al., 2004) or even earlier. In our own experience, most of the housed APP/PS1 mice indeed developed AD features at 6 months of age, and thus the time point at 5 months of age was selected for this study, since we aimed to examine the accelerating development of AD by SEVO, and the SEVO-induced behavioral disorder and pathological changes at 6 months of age may be concealed or weakened due to the automatic disease progression.

The detrimental effects of SEVO on neuronal cell integrity have been proposed in some previous studies (Morris et al., 1982; Burgess et al., 2002; Criswell et al., 2004; Fang et al., 2012). Very recently, Chikusetsu saponin IVa was used to alleviate SEVO-induced neuroinflammation and cognitive impairment by blocking NLRP3, likely through reducing apoptosis of neuronal cells (Shao et al., 2020). In another research, Geng et al. (2018) elegantly showed that SEVO-induced Amyloid pathology and spatial learning impairment in APP/PS1 Mice can be reversed by control of autophagy. Moreover, autophagic pathway has also been nicely shown to be involved in the epigenetic regulation of NLRP3 in the SEVO-induced mouse behavioral impairment in Fang et al. (2021). However, the exact mechanisms were still unknown, especially the involvement of BDNF. BDNF is a key neurotrophic factor that promotes synaptic plasticity, as well as the survival and function of neuronal cells (Aarons et al., 2019; Bomba et al., 2019; Fransquet et al., 2020). It has been shown to play a pivotal role in neurodegeneration. However, whether BDNF is a critical factor in the SEVO-regulated NLRP3 activation to induce neurodegeneration has not been investigated previously.

We found evidence of suppression of BDNF by NLRP3 inflammasome in some previous reports. First, NLRP3 was shown as critical immune sensors that causally link systemic inflammation to aging and cognitive disorder (Youm et al., 2013). In this study, ablation of NLRP3 inflammasome protected mice from age-related increases in the innate immune activation, alterations in central nervous system (CNS) transcriptome, and astrogliosis (Youm et al., 2013). Interestingly, IL-1 was found to mediate NLRP3 inflammasome-dependent improvement in cognitive function and motor performance in aged mice, in which BDNF was likely involved (Youm et al., 2013). Second, gastrodin, which possesses specific anti-oxidative, anti-inflammatory, and neuroprotective effects, was shown to ameliorate diabetes-associated cognitive dysfunction (Ye et al., 2018). Intriguingly, this study showed that gastrodin increased the expression of BDNF and decreased the activation of NLRP3 inflammasome simultaneously (Ye et al., 2018). Finally, in a model of brain ischemia, Ward et al. (2019) nicely showed that BDNF was reduced by NLRP3 inflammasome. These studies also showed a likely negative relationship between BDNF and NLRP3 but did not demonstrate a causative link, especially in the SEVO-mediated neurodegeneration.

Previous studies have mainly focused on the role of NLRP3 inflammasome in microglia as well as its effects on neurodegenerative diseases in APP/PS1 mice (Heneka et al., 2013) and in humans (Haque et al., 2020). However, NLRP3 has also been detected in human neurons, suggesting that neuron-derived NLRP3 also play a role in the neuroinflammation (Von Herrmann et al., 2018; He et al., 2020). In this study, in isolated neuronal cells from APP/PS1, we further detected the expression of NLRP3. The high expression of NLRP3 in microglia and their potential role as resident innate immune cells of the CNS in the earliest local response to the injury or infection makes microglia the initial responders for SEVO, in which their differentiation and polarization status may change after activation of the NLRP3 inflammasome machinery; the activation of NLRP3 in neurons may reflect a response to the stress from the microenvironment to determine the survival or death of the neuronal cells. This NLRP3 activation in neurons may occur later than the NLRP3 activation in microglia. It is noteworthy that the observed effects of SEVO in the current study may result from a combined or synergistic effect on neurons and microglia, in a NLRP3-dependent manner. It is even possible that the activation of NLRP3 in microglia may in turn activate NLRP3 signaling in neurons through IL-1β and IL-18 in a paracrine manner. It may be interesting to use neuron-specific NLRP3 knockout mice and microglia-specific NLRP3 knockout to determine the interaction between the two types of cells in terms of NLRP3 activation in a future study.

Moreover, NLRP3 inflammasome appeared to be an upstream regulator for BDNF, shown in a loss-of-function experiment using a specific inhibitor of NLRP3. MCC950 has been widely used as a specific inhibitor of NLRP3 (Coll et al., 2019; Marin-Aguilar et al., 2019; Tapia-Abellan et al., 2019; Wu et al., 2020). Moreover, we showed that this regulatory axis “NLRP3/BDNF” may be the key signal pathway that is responsible for the SEVO-mediated neurotoxicity. Thus, addressing and suppressing activation of NLRP3 inflammasome may be an attractive novel strategy to prevent SEVO-associated neurotoxic effects in clinical application.

## Data Availability Statement

The original contributions presented in the study are included in the article/supplementary material, further inquiries can be directed to the corresponding author/s.

## Ethics Statement

The animal study was reviewed and approved by the Second Hospital of Shandong University.

## Author Contributions

GL, YW, FC, DW, LZ, and YJ are responsible for data acquisition and analysis. GL and YJ are responsible for study conception and design and data acquisition and analysis. YJ was responsible for funding, manuscript writing and is the guarantor of the study. All authors agreed with the final version of the manuscript to be published.

## Conflict of Interest

The authors declare that the research was conducted in the absence of any commercial or financial relationships that could be construed as a potential conflict of interest.

## Publisher’s Note

All claims expressed in this article are solely those of the authors and do not necessarily represent those of their affiliated organizations, or those of the publisher, the editors and the reviewers. Any product that may be evaluated in this article, or claim that may be made by its manufacturer, is not guaranteed or endorsed by the publisher.
